# Supplementary material for: Continuous-wave upconversion lasing with a sub-10 W cm−2 threshold enabled by atomic disorder in the host matrix
Source: Nat Commun. 2021 Jul 21;12:4437. doi: 10.1038/s41467-021-24751-z (PMC8295256; doi:10.1038/s41467-021-24751-z)
Supplement: Supplementary file 1 — Supplementary information [file 41467_2021_24751_MOESM1_ESM.pdf]

Supplementary Information

Continuous-wave upconversion lasing with a sub-10 W cm^{-2} threshold enabled by atomic disorder in the host matrix

Byeong-Seok Moon[1], Tae Kyung Lee[2,3], Woo Cheol Jeon[2], Sang Kyu Kwak[2], Young-Jin Kim[4,*] and Dong-Hwan Kim[1,5*]

[1]School of Chemical Engineering, Sungkyunkwan University, Suwon 16419, Republic of Korea

[2]Department of Energy Engineering, School of Energy and Chemical Engineering, Ulsan National Institute of Science and Technology (UNIST), Ulsan 44919, Republic of Korea

[3]Photovoltaics Research Department, Korea Institute of Energy Research (KIER), Daejeon 34129, Republic of Korea

[4]Department of Mechanical Engineering, Korea Advanced Institute of Science and Technology (KAIST), Daejeon 34141, Republic of Korea

[5]Biomedical Institute for Convergence at SKKU (BICS), Sungkyunkwan University, Suwon 16419, Republic of Korea

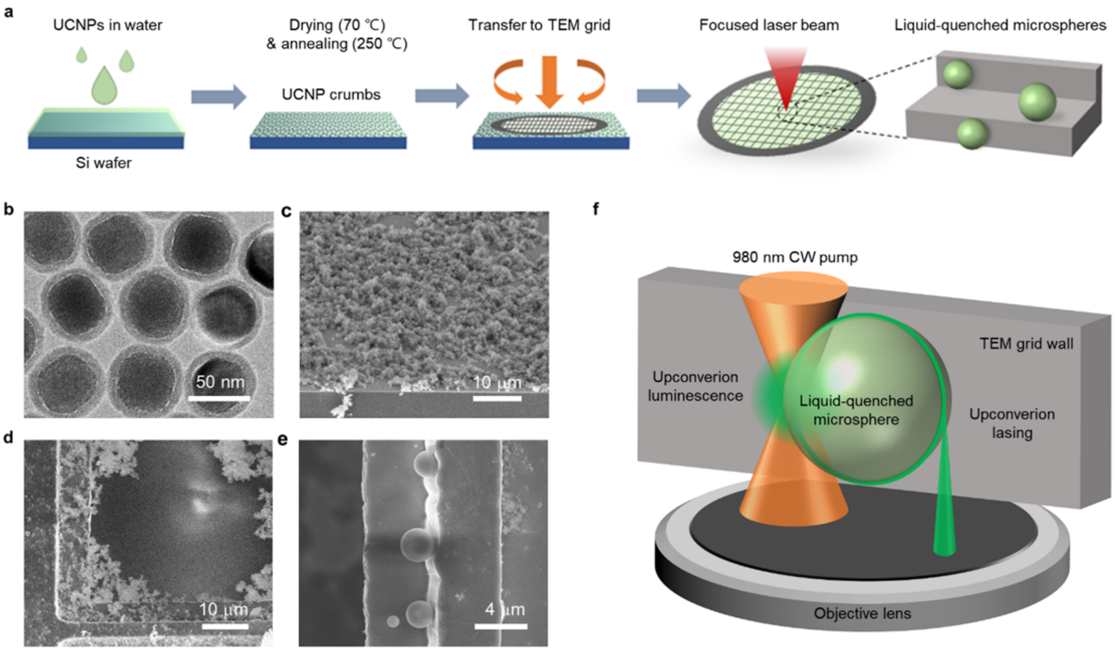

Supplementary Figure 1. a. Fabrication schematic for the liquid-quenched upconversion microspheres (LQUM). **b.** As-synthesized upconversion nanoparticles (UCNPs). Note that the incorporation of a thin SiO_2 layer is essential to realize complete amorphization after the liquid-quenching process[1]. **c.** UCNP crumbs on a Si wafer after drying and annealing. **d,** UCNP crumbs on the TEM grid after the transfer process. **e,** As-synthesized LQUMs on the TEM grid. **f,** Simplified optical setup for the observation of LQUM microlasers.

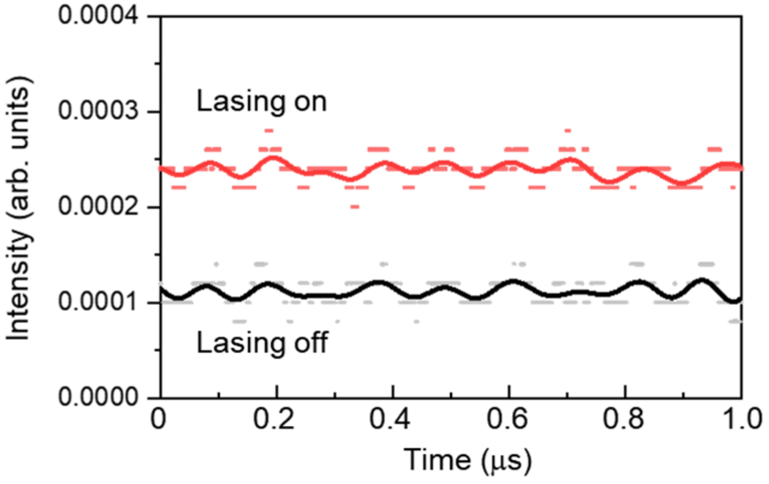

Supplementary Figure 2. Continuous-wave laser operation. Temporal stability analysis reveals that the continuous-wave laser operation is stable within the instrumental noise level. The emission was measured by a Si-APD (Thorlabs, APD410A) and recorded with an oscilloscope (Rigol, DS2102A-S).

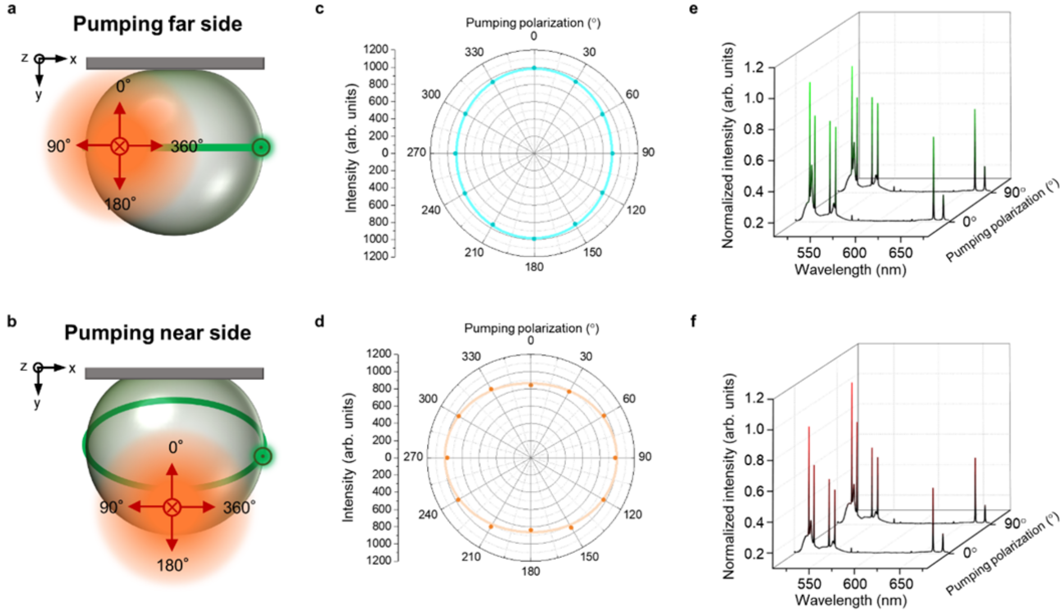

Supplementary Figure 3. Pumping polarization effect. a-b. Two cases of pumping depending on the pumping side: pumping far (**a**) and near (**b**) from the laser emission point. **c-d**. Polar plot of the intensity of laser emission vs the pumping polarization angle in the xy plane for pumping at the far (**c**) and near (**d**) sides. **e-f**. Emission spectrum with the pumping polarization angle in the xy plane (0° and 90°) for pumping at the far (**e**) and near (**f**) sides. By controlling the pumping polarization angle, we confirmed that the laser emission exhibits negligible sensitivity to the pumping polarization.

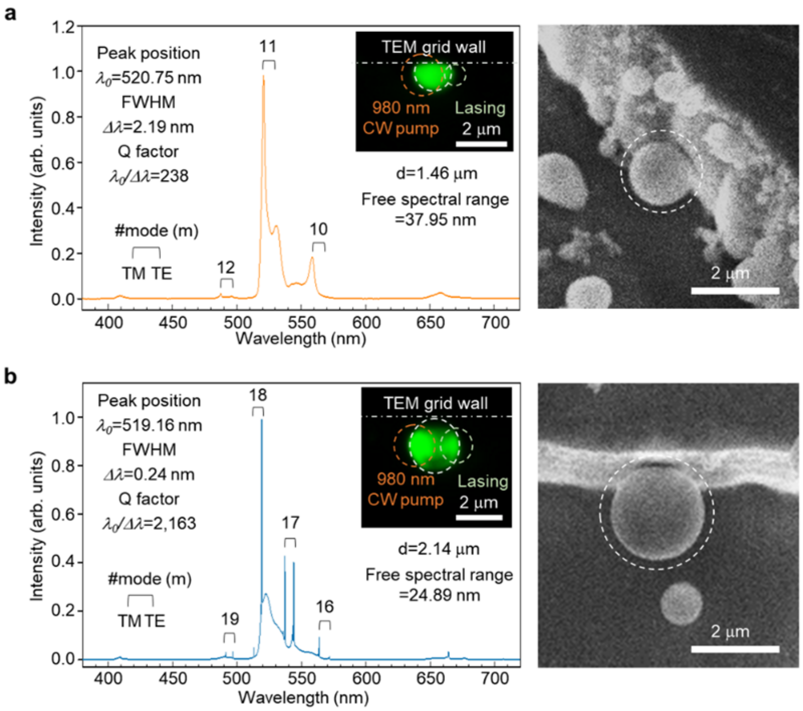

Supplementary Figure 4. Study of the size of liquid-quenched upconversion microspheres (LQUMs). a-b. Emission spectrum of upconversion lasing from LQUMs of various diameters. An LQUM of 1.46 μm in diameter (**a**) exhibits a low Q factor due to high curvature loss[2]. An LQUM of 2.14 μm in diameter (**b**) does not exhibit resonance with the red emission band of Er^{3+}; therefore, upconverted photons of the red emission band could not participate in the laser process. To maximize the laser efficiency, we concluded that 2.44 μm is the optimal LQUM diameter in the present study, which is in line with a theoretical calculation[3].

Supplementary Note 1

We find that the efficiency of the upconversion microlaser is determined by three factors: (1) heat generation in the microcavity, (2) effective pump power, which contributes to the upconversion luminescence, and (3) the efficiency of pump-to-gain coupling. All the factors are optimized by the positional relationship of the Gaussian-type free-space pumping beam and the microcavity.

To prove these relationships, we investigated the laser intensity with various pump positions: the edge, the rim and the center of the microsphere (see the pump positions marked in the inset of Figure 1d and Supplementary Figure 5). At high pumping power (above 1.0 MW cm^{-2}), the most intense laser outputs were observed when the very edge of the LQUM was pumped by virtue of the minimal heat generation interfering with the stable lasing; the center and the rim pumpings showed inferior laser intensities despite the upconversion luminescence intensities being stronger there than at the edge (Supplementary Figure 5). Depending on the pumping position, i.e., the edge, the rim and the center (Supplementary Figure 5a), the upconversion luminescence and the upconversion lasing intensities varied significantly (Supplementary Figure 5b). First, the heat generation for the rim and the center pumping was significantly higher than that for the edge pumping, which was supported by the laser line shifts (which originated from the increased temperature of the microsphere) (Supplementary Figure 5c). On the other hand, compared with the other pumping positions, the edge pumping results in severe pump power loss due to its minimal overlap with the pump beam, indicated by the weakest upconversion luminescence coming from edge pumping (Supplementary Figure 5d). Additionally, the low intensity ratio of $^2H_{11/2}\rightarrow{}^4I_{15/2}$ to $^4F_{9/2}\rightarrow{}^4I_{15/2}$ transitions supports that the pump power loss is more significant in edge pumping than in the other pumpings[1]. The pump power loss for the edge pumping is also

responsible for the low intensity ratio of $Peak_H$ to $Peak_F$ (Supplementary Figure 5e). Nevertheless, the highest laser peak integrated intensity was found for the edge pumping. Therefore, at high pumping power, detrimental heat generation is more crucial than the loss of pump power for intense upconversion lasing.

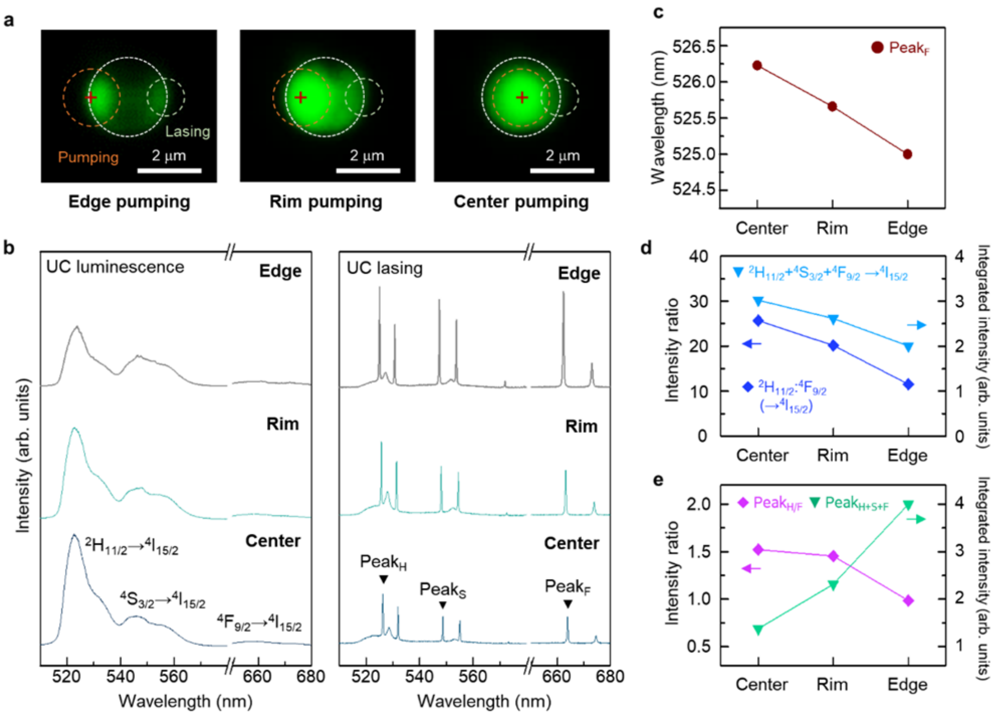

Supplementary Figure 5. Upconversion luminescence and upconversion lasing depend on the pumping position. a. Color images of an LQUM depending on the pumping position: edge, rim and center pumping at a high pumping power density of 3.16 MW cm^{-2}. **b**. Emission spectra of upconversion luminescence and upconversion lasing depending on the pumping position. **c**. The central wavelength of $Peak_F$ as a function of pumping position. **d-e**. Comparison of upconversion luminescence (**d**) and upconversion lasing (**e**) in terms of intensity ratio and integrated intensity depending on the pumping position.

However, as we decreased the pumping power to less than 1.0 MW cm^{-2}, where the

heat effects are less significant, rim pumping showed the most sustainable lasing, which led to a lower lasing threshold than the edge and center pumping by a factor of 28 (Supplementary Figure 6). This is an unexpected result because most papers have reported edge pumping for optimal laser performance. As the pump power density decreases, the integrated lasing intensity of the edge and the center pumping systems collapsed faster than that of the rim pumping system (Supplementary Figure 6a). As a result, the most sustained lasing was produced by rim pumping (Supplementary Figure 6b, c). The lasing intensity of the center pumping collapsed rapidly due to it resulting in the weakest pump-to-gain coupling efficiency[4] despite the minimum power loss. On the other hand, the edge pumping resulted in the fastest collapse of the laser intensity when the pump power decreased, which is attributed to its highest power loss. This loss occurred because the power loss out of the microsphere fatally hinders the population inversion of the activator's upconversion lasing states through depletion of the intermediate states of the neighboring dopants. Therefore, we concluded that rim pumping provides not only a strong pump-to-gain coupling effect but also a low pump power loss, leading to the most sustainable upconversion lasing at low pump power excitation, where the heating effect is negligible.

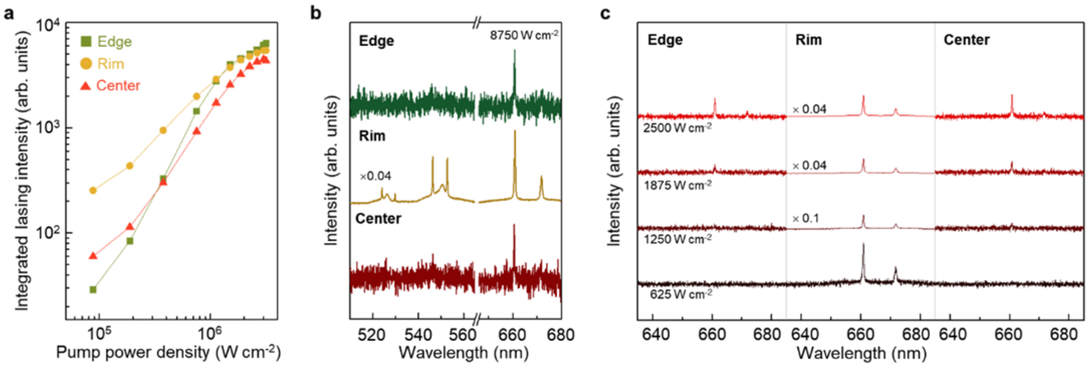

Supplementary Figure 6. Investigation of upconversion lasing with low pump power density. a. The power dependence of upconversion lasing on the pumping position. **b-c**. The emission spectrum of upconversion lasing

peaks depending on the pumping position at 8,759 W cm^{-2} (**b**) and below 2,500 W cm^{-2} (**c**). The lasing thresholds were above ~1.2 kW cm^{-2} in the cases of edge pumping and center pumping.

To support the fact that our monolithically designed WGM resonator yields sufficient pump-to-gain interactions with a free-space beam at the rim pumping, the pump-to-gain interactions have been carefully studied in terms of the pump position through numerically simulated spatial overlaps between the optical field distributions of the transverse magnetic (TM) mode at 660 nm in an LQUM of 2.44 μm in diameter and a single Gaussian-type free-space pump beam (λ=980 nm, N.A.=1.3). The simulation results show that the most efficient pump-to-gain coupling is attained at the rim of the LQUM, where ~21% of the microsphere's radius is far from the surface of the microsphere (Supplementary Figure 7), which is in line with our experimental observations (Supplementary Figure 6).

To investigate the pump-to-gain coupling effect in the LQUM with a 2.44-μm diameter, we simulated the overlap of the pumping beam and the optical mode in the gain medium, as shown in Supplementary Figure 7a, following three approximations.

1) A Gaussian-type pump beam irradiates the microsphere along the z-axis.
2) The pump beam moves on the focal plane (xy plane), which includes the maximum intensity point of the pump beam and the edge and the center of the simulated microsphere.
3) The overlap area of the pump beam and the optical mode in the cross-section (xz plane) represents the pump-to-gain coupling effect in the microsphere.

In a real situation, the pump beam intensity does not follow a perfect Gaussian beam profile because diode lasers are not a point light source. The distributed emitting points in the active area of several micrometers across broaden the focusing spot size to be larger than the

ideal Gaussian beam waist, which is determined by the numerical aperture of the focusing lens[5]. Furthermore, astigmatism, which refers to the difference in the focusing distance between the fast and slow axes of the active area, produces the larger focusing spot size at the focal plane[6]. For these reasons, the focused spot of our optical setup is approximated to be 3 μm in diameter. Nevertheless, because the pump beam profile in real situations can be considered the sum of coherent lights from multiple point sources, we were able to investigate the pump-to-gain coupling effect using the ideal Gaussian-type beam profile of our optical pumping setup ($2\omega_0$=720 nm for λ=980 nm, N.A.=1.3). As a side note, we considered only the two-dimensional pump-to-gain coupling effect rather than the three-dimensional case because most coupling effects occur on the circumferential plane where the resonance occurs[4], in our case, the xz plane.

To determine the optical mode of the upconversion lasing at 660 nm (TM mode, m=15, n=1.719), we computed the TM mode optical field distribution in the microsphere using MATLAB software following methods in the literature[7], as shown in Supplementary Figure 7b. To quantify the pump-to-gain interaction, we newly defined the effective pump-to-gain coupling distance, which is defined as the full-width-half-maximum of the optical fields from the WGM resonator's surface for effective coupling of gain with pump laser beam, was determined to be 370 nm. Next, we defined the pump-to-gain coupling effect as the magnitude of the effective pump power that is converted into upconversion lasing. Since the upconversion at 660 nm (two photon excitation) is proportional to the pump power intensity to the second power ($\propto I^2$), we calculated the pump-to-gain coupling effect as the sum of the square of the pump intensity that overlaps with the effective pump-to-gain coupling distance. With these conditions, we plotted the normalized pump-to-gain coupling effect depending on the pumping positions from the edge to the center (Supplementary Figure 7c). A considerable

magnitude of the pump-to-gain coupling effect is observed at the very edge (Supplementary Figure 7d). As the pump moves toward the center, the maximum coupling effect was found at 21% of the radius from the edge (Supplementary Figure 7e), strongly supporting our experimental observations (Supplementary Figure 6). Exceeding the maximum point, the pump-to-gain coupling effect continued to dramatically decrease, reaching a value smaller than one-seventh of the maximum for center pumping (Supplementary Figure 7f).

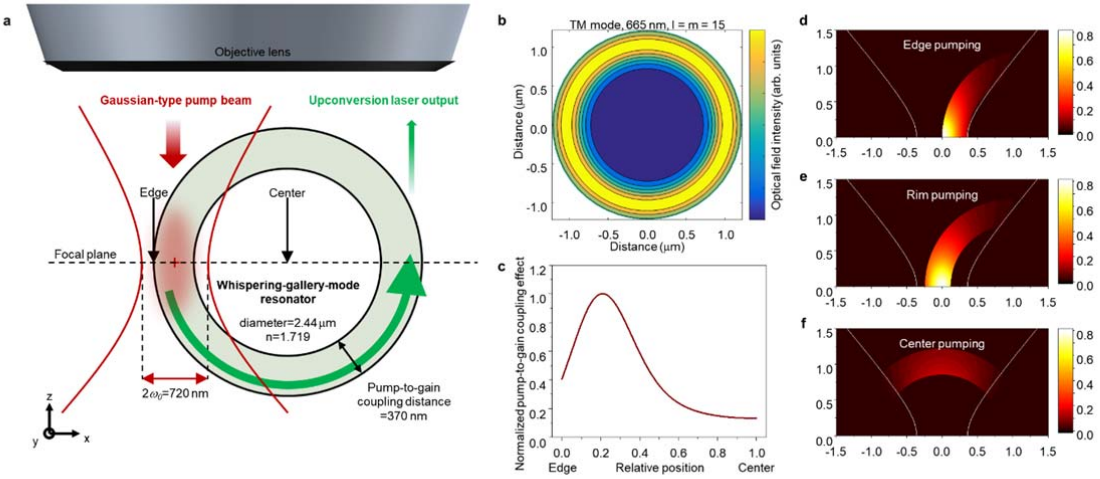

Supplementary Figure 7. The pump-to-gain coupling effect depending on the pump position. a. Schematic of a pump-to-gain coupling effect simulation. **b**. Optical field distribution of the microsphere. **c**. The normalized pump-to-gain coupling effect depending on the pumping position from the edge (0.0) to the center (1.0). **d-f**. The simulated pump power distribution depending on the pumping position, i.e., if it is at the edge (**d**), the rim (**e**), or the center (**f**). White lines indicate the Gaussian beam radius.

For the WGM resonators with micrometer dimensions, the pump position optimization to maximize the pump-to-gain interactions becomes central (Supplementary Figure 8). Using the approximations in Supplementary Figure 7, we further simulated the pump-to-gain coupling effect with microspheres of various sizes. As the microsphere size decreases, the position of the maximum pump-to-gain coupling moves from the edge to the center (Supplementary Figure 8a), while the coupling magnitude decreases (Supplementary Figure 8b). Therefore, to maximize the intrinsically weak pump-to-gain coupling effect, the pumping position needs to be precisely optimized, particularly when the microsphere sizes are comparable to the size of the pump beam (*i.e.*, microspheres smaller than ~4 μm in diameter) (Supplementary Figure 8c).

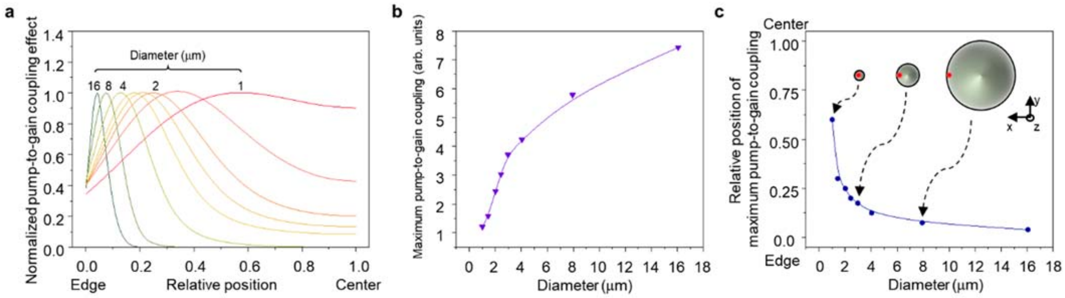

Supplementary Figure 8. Pump-to-gain coupling effect as a function of microsphere size. a. The normalized pump-to-gain coupling effect depending on the microsphere size. The magnitude of the pump-to-gain coupling effect represents the effective pump power that is converted into upconversion lasing (the sum of the square of the pump intensity that overlaps with the pump-to-gain coupling distance as we calculated in Supplementary Figure 7). The maximum magnitude point was normalized to 1 for fair comparison. **b**. The maximum value of the pump-to-gain coupling effect depending on the microsphere size. **c**. The relative pumping position of the maximum pump-to-gain coupling effect depending on the microsphere size.

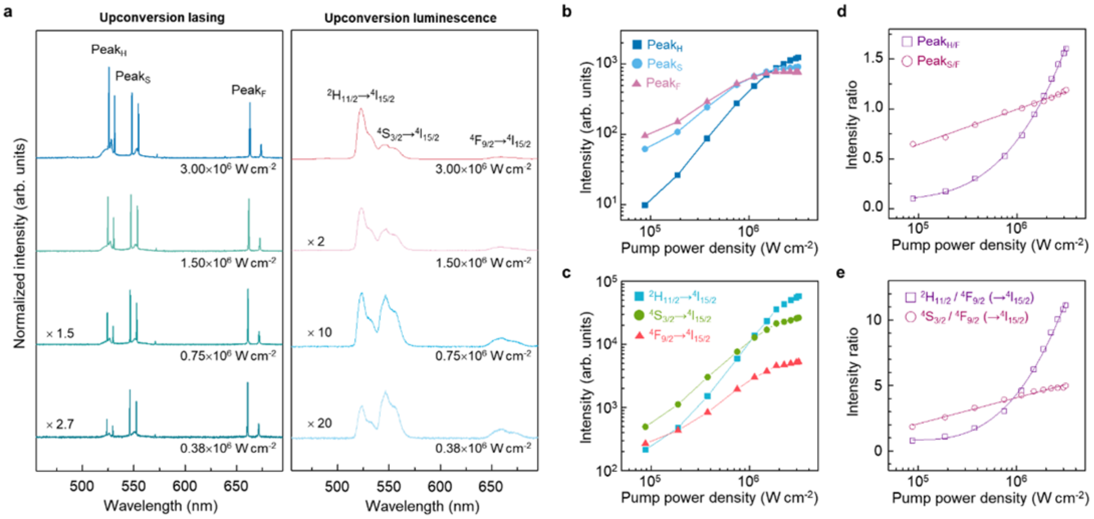

Supplementary Figure 9. Power dependence of upconversion lasing. **a**. Comparison of upconversion lasing and upconversion luminescence at different pump power densities. **b-e**. Power dependence of the intensities and the intensity ratios of upconversion lasing at the observation spot (**b**) and upconversion luminescence at the pumping spot (**c**). Above the lasing thresholds, the upconversion lasing intensity increases proportionally with the upconversion luminescence intensity of the corresponding emission bands. This finding is confirmed by the identical intensity ratios of $Peak_H/Peak_F$ and $^2H_{11/2}/^4F_{9/2}$ ($\rightarrow^4I_{15/2}$) in upconversion lasing (**d**) and upconversion luminescence (**e**), respectively.

Supplementary Note 2

Computational details

Modeling of amorphous $NaYF_4$+SiO_2 structure

To construct liquid-quenched amorphous $NaYF_4$ with SiO_2[1], we first performed an all-atom molecular dynamics (AAMD) simulation by using the bulk model system of $(NaYF_4SiO_2)_{600}$ (**Supplementary Figure 10**). Nonbonded interaction parameters in the universal force field (UFF)[8] were used for van der Waals interactions, and the formal charges of each atom were assigned for electrostatic interactions. For van der Waals interactions, the atom-based summation method was used with a cutoff distance of 12.5 Å. For electrostatic interactions, the Ewald summation method was used with an Ewald accuracy of 0.001 kcal/mol. The *NVT* (*i.e.,* canonical; constant temperature) ensemble was applied with a Nose thermostat[9]. AAMD simulations were performed in the following order to mimic the synthesis of LQUMs. First, an AAMD simulation was conducted for 500 ps at 298 K for initial relaxation. Then, the AAMD simulation was heated for 2000 ps at 2273 K and cooled for 200 ps at each temperature as the temperature was decreased to 298 K in 100 K intervals, which represents liquid quenching by the laser in the experiment. Finally, the AAMD simulation was conducted for 2000 ps at 298 K for relaxation. The time step of the AAMD simulation was 1 fs. All AAMD simulations were performed by using Materials Studio 2019[10].

After performing the AAMD simulation, density functional theory (DFT)-based *ab initio* molecular dynamics (AIMD) simulations were performed to construct a more valid amorphous system. To perform the AIMD simulation, amorphous configurations of $NaYF_4$+SiO_2 were randomly extracted from the bulk model system obtained by the AAMD simulation (**Supplementary Figure 10**) based on the interatomic distances of each pair from the radial distribution function (RDF). Ten types of amorphous $NaYF_4$+SiO_2 model systems

were constructed with a 1:1 volume ratio, which was the ratio for the experimental LQUM (**Supplementary Figure 11**). The Yb^{3+}-, Er^{3+}- and Tm^{3+}-doped systems were constructed by substitution of one Y atom in each model system (**Figures S12, S13, and S14**). All AIMD simulations were carried out using the CASTEP program[10,11]. The Perdew-Burke-Ernzerhof (PBE) functional within the generalized gradient approximation (GGA)[12] was used for the exchange-correlation functional. Spin-polarized calculations were performed. A norm-conserving pseudopotential was used[13] with a 780-eV energy cutoff. The Brillouin zone was sampled by 1 × 1 × 1 *k*-point grids with the Monkhorst-Pack[14] for the amorphous model systems. The tolerance of the self-consistent field calculation was 2×10^{-6} eV $atom^{-1}$. The smearing was set to 0.5 eV. Nose[9] and Andersen[15] were used for thermostat and barostat, respectively. For the AIMD simulations, an *NPT* simulation was first performed for 10 ps at 298 K and 1 atm. After that, an *NVT* simulation was performed for 10 ps at 298 K. The time step of the AIMD simulation was 1 fs. After the AIMD simulations, the phonon density of states (phonon DOS) of amorphous systems were calculated. For the calculation of phonon DOS, DFT calculations were performed by using the finite displacement method[16]. The supercell was defined by a cutoff radius of 5 Å. The Broyden–Fletcher–Goldfarb–Shanno (BFGS) algorithm[17] was applied to geometry optimizations of amorphous systems. The convergence tolerances of energy, force, stress, and displacement for the geometry optimizations were set to 2×10^{-5} eV $atom^{-1}$, 0.05 eV $Å^{-1}$, 0.1 GPa, and 0.002 Å, respectively. The *k*-points used for the phonon DOS were 2 × 2 × 2.

Hexagonal crystal $NaYF_4$ structure

To compare with the phonon DOS of the amorphous structure, we performed DFT calculations on hexagonal crystal $NaYF_4$ structures (hexagonal $NaYF_4$, Yb^{3+}-doped

hexagonal $NaYF_4$, Er^{3+}-doped hexagonal $NaYF_4$, and Tm^{3+}-doped hexagonal $NaYF_4$ in **Supplementary Figure 15**). For the calculations of the phonon DOS of hexagonal $NaYF_4$, Yb^{3+}-doped hexagonal $NaYF_4$, Er^{3+}-doped hexagonal $NaYF_4$, and Tm^{3+}-doped hexagonal $NaYF_4$ systems, the finite displacement method with cell optimization was performed with the same settings as the amorphous system except for the *k*-point sets, which were 2 × 2 × 2 (cell optimization) and 4 × 4 × 3 (phonon DOS).

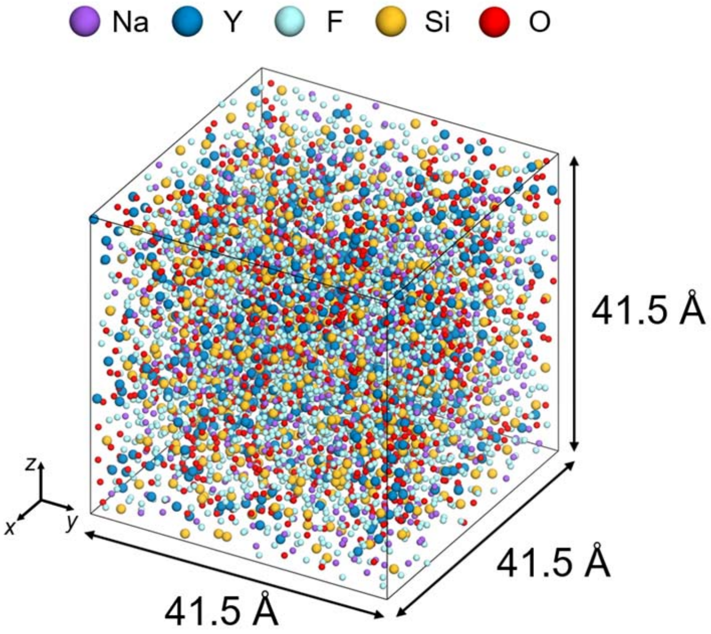

Supplementary Figure 10. **Model system of bulk $(NaYF_4SiO_2)_{600}$.** The number of atoms is 5,400 in this system. The angles of the system are 90°.

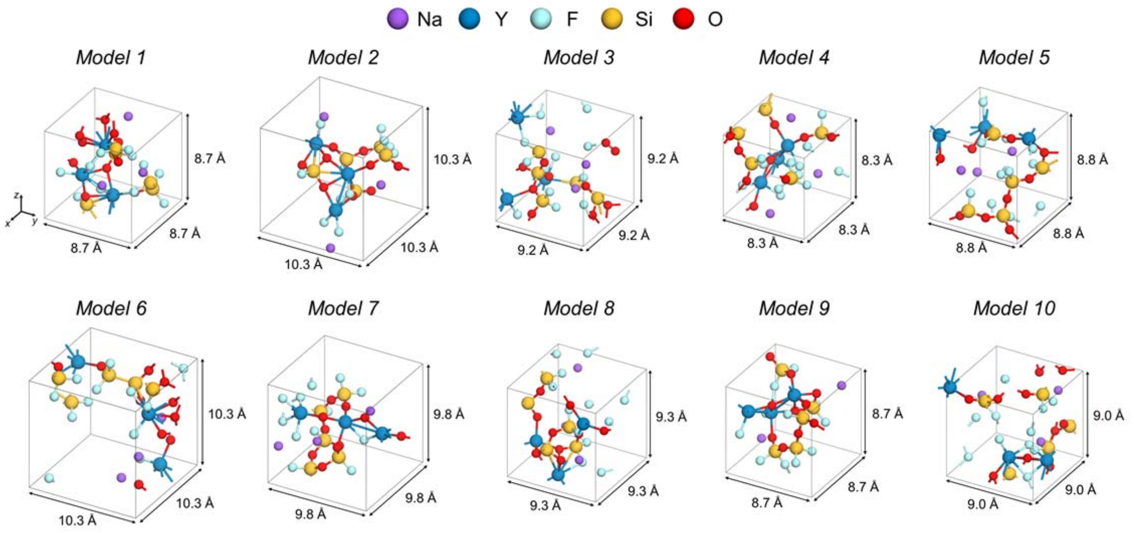

Supplementary Figure 11. Model systems of amorphous $(NaYF_4)_3(SiO_2)_5$. The number of atoms is 33 in this system. The angles of all systems are 90°.

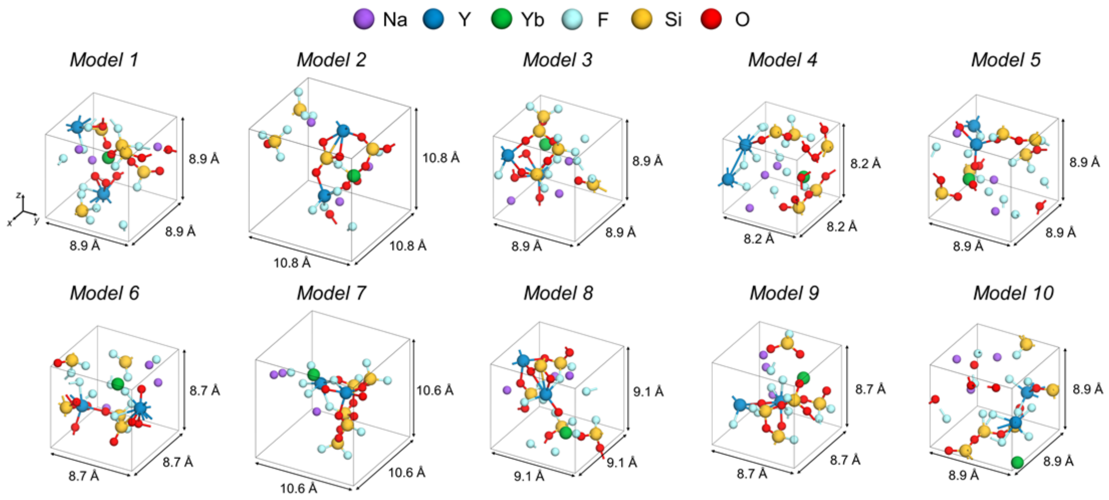

Supplementary Figure 12. **Model systems of amorphous $(Na_3Y_2YbF_{12})(SiO_2)_5$.** The number of atoms is 33 in this system. The angles of all systems are 90°.

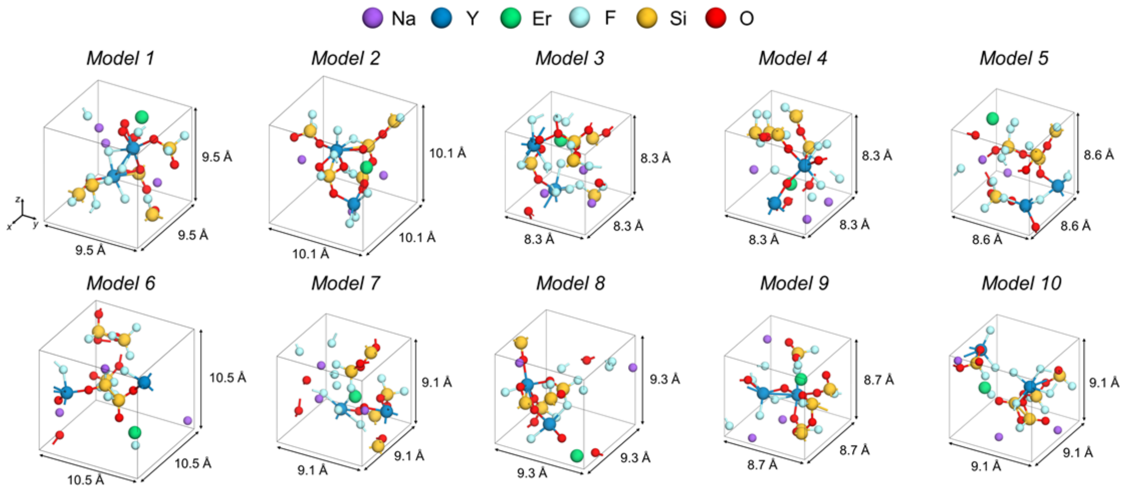

Supplementary Figure 13. Model systems of amorphous $(Na_3Y_2ErF_{12})(SiO_2)_5$. The number of atoms is 33 in this system. The angles of all systems are 90°.

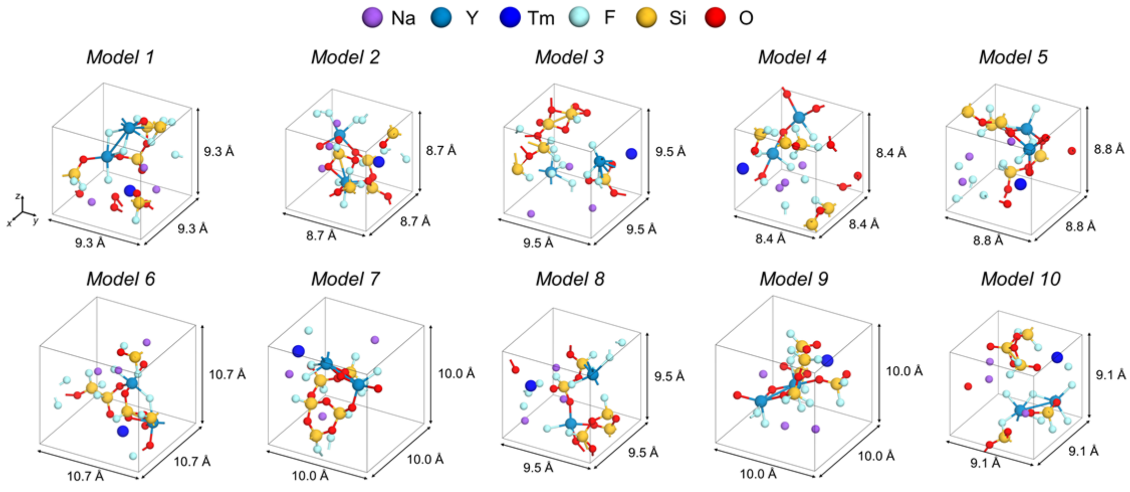

Supplementary Figure 14. Model systems of amorphous $(Na_3Y_2TmF_{12})(SiO_2)_5$. The number of atoms is 33 in this system. The angles of all systems are 90°.

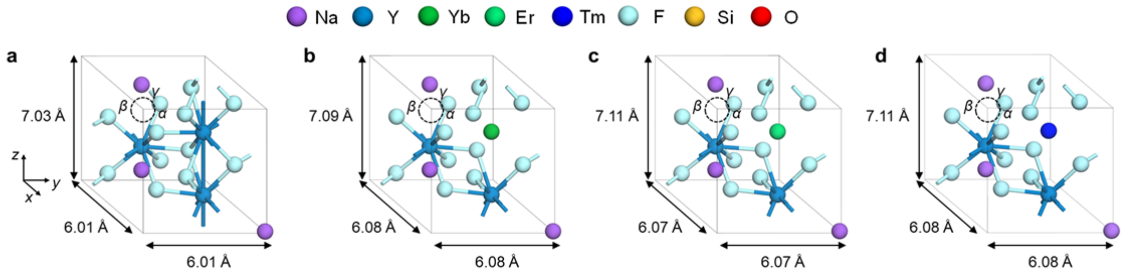

Supplementary Figure 15. Model systems of the hexagonal crystal $NaYF_4$ structure. a-d. Hexagonal crystal $NaYF_4$ (**a**) and Yb-doped (**b**), Er-doped (**c**), and Tm-doped (**d**) hexagonal crystal $NaYF_4$ structures. The number of atoms is 18 in each system. The angles of all systems are 90° (α), 90° (β), and 120° (γ).

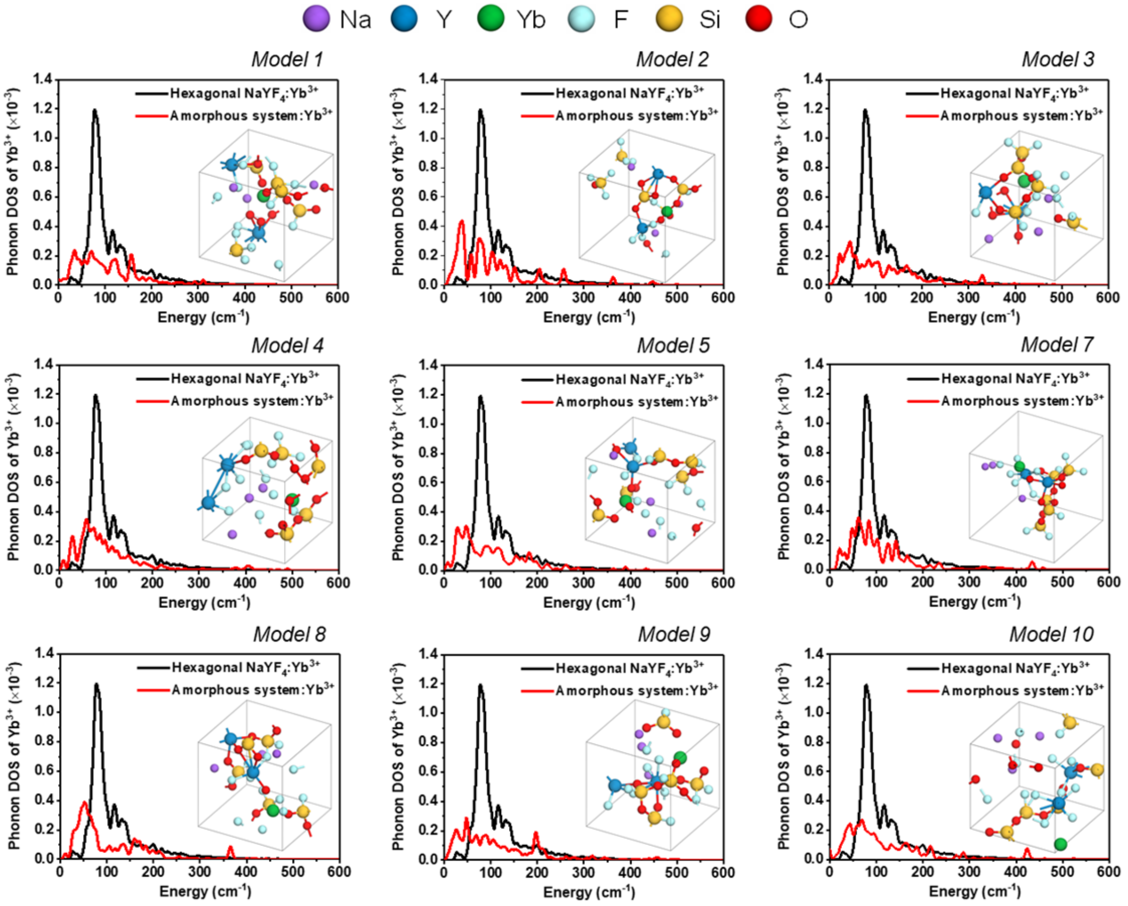

Supplementary Figure 16. Phonon DOS of Yb^{3+} in Yb-doped hexagonal crystal $NaYF_4$ and Yb-doped amorphous systems. Note that the models of Yb-doped amorphous systems and Yb-doped hexagonal crystal $NaYF_4$ are shown in Supplementary Figure 12 and Supplementary Figure 15b, respectively.

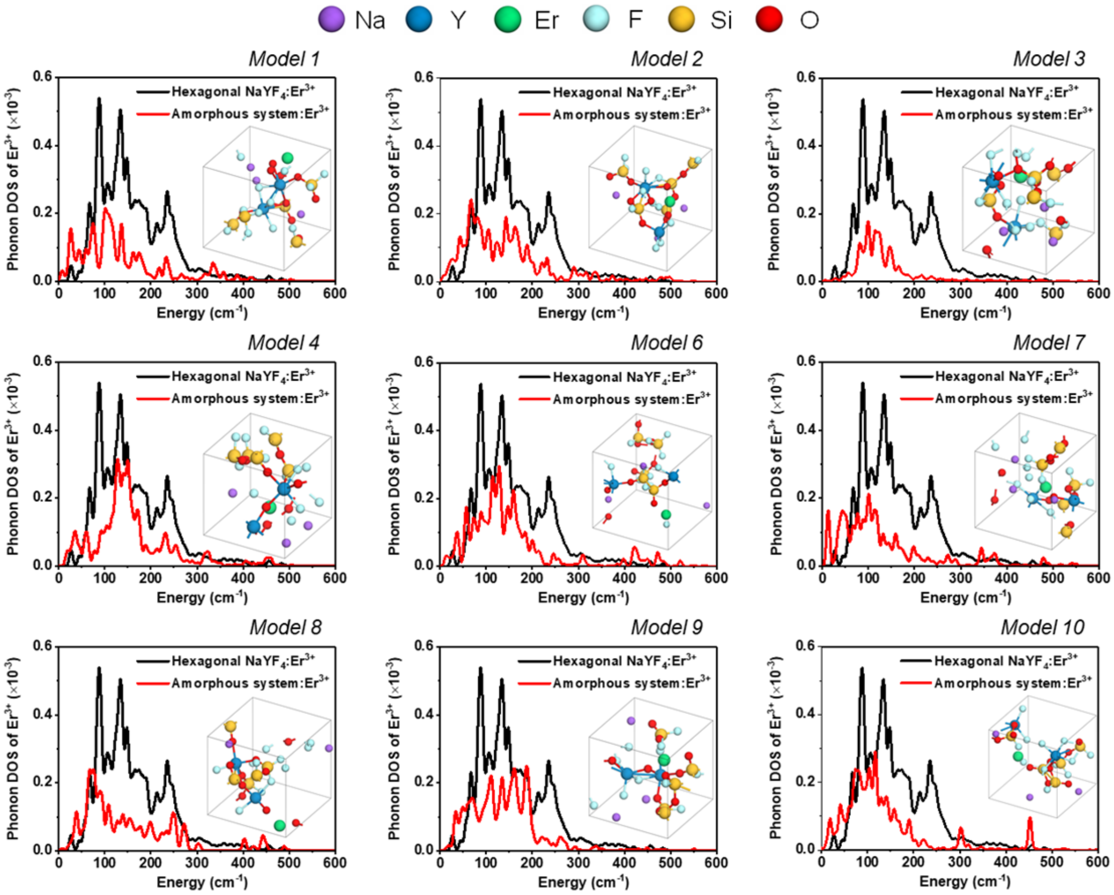

Supplementary Figure 17. Phonon DOS of Er^{3+} in Er-doped hexagonal crystal $NaYF_4$ and Er-doped amorphous systems. Note that the models of Er-doped amorphous systems and Er-doped hexagonal crystal $NaYF_4$ are shown in Supplementary Figure 13 and Supplementary Figure 15c, respectively.

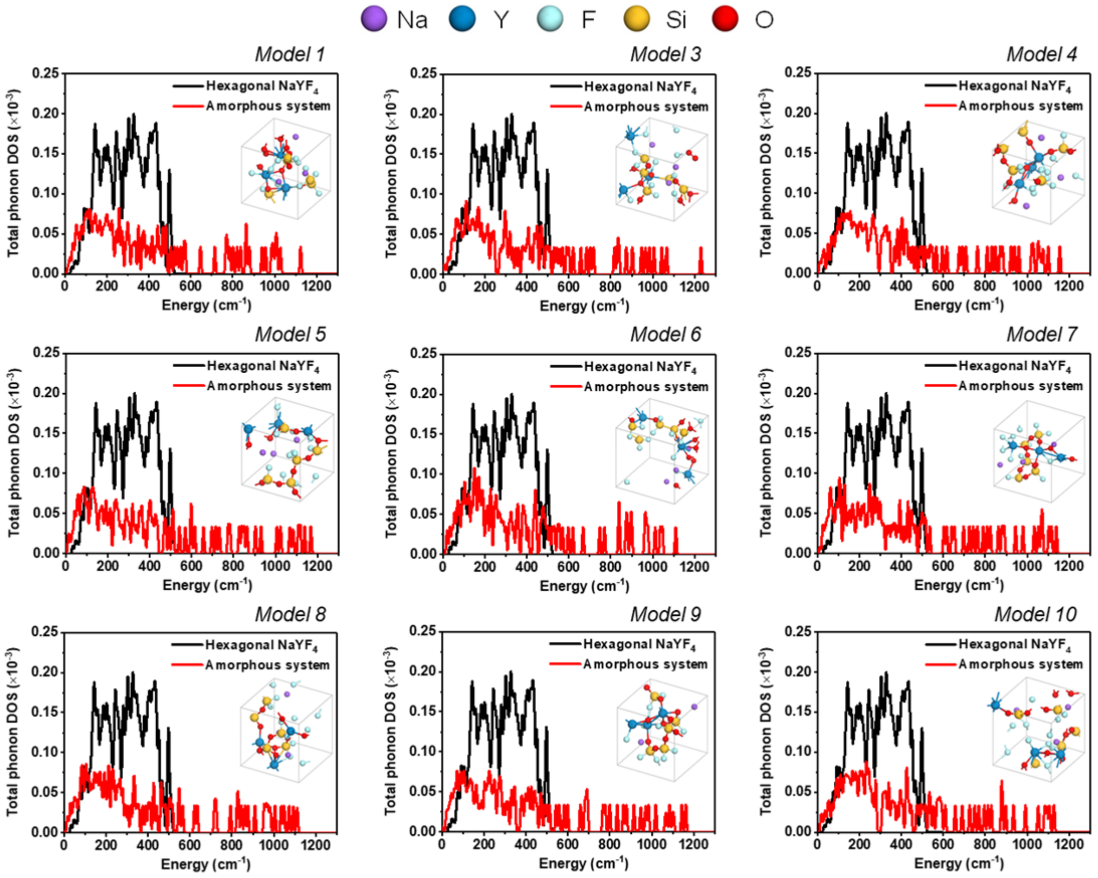

Supplementary Figure 18. Total phonon DOS of hexagonal crystal $NaYF_4$ and amorphous systems. Note that models of amorphous systems and hexagonal crystal $NaYF_4$ are shown in Supplementary Figure 11 and Supplementary Figure 15a, respectively. Note that the results of the total phonon DOS are normalized by dividing by the total number of atoms in each system.

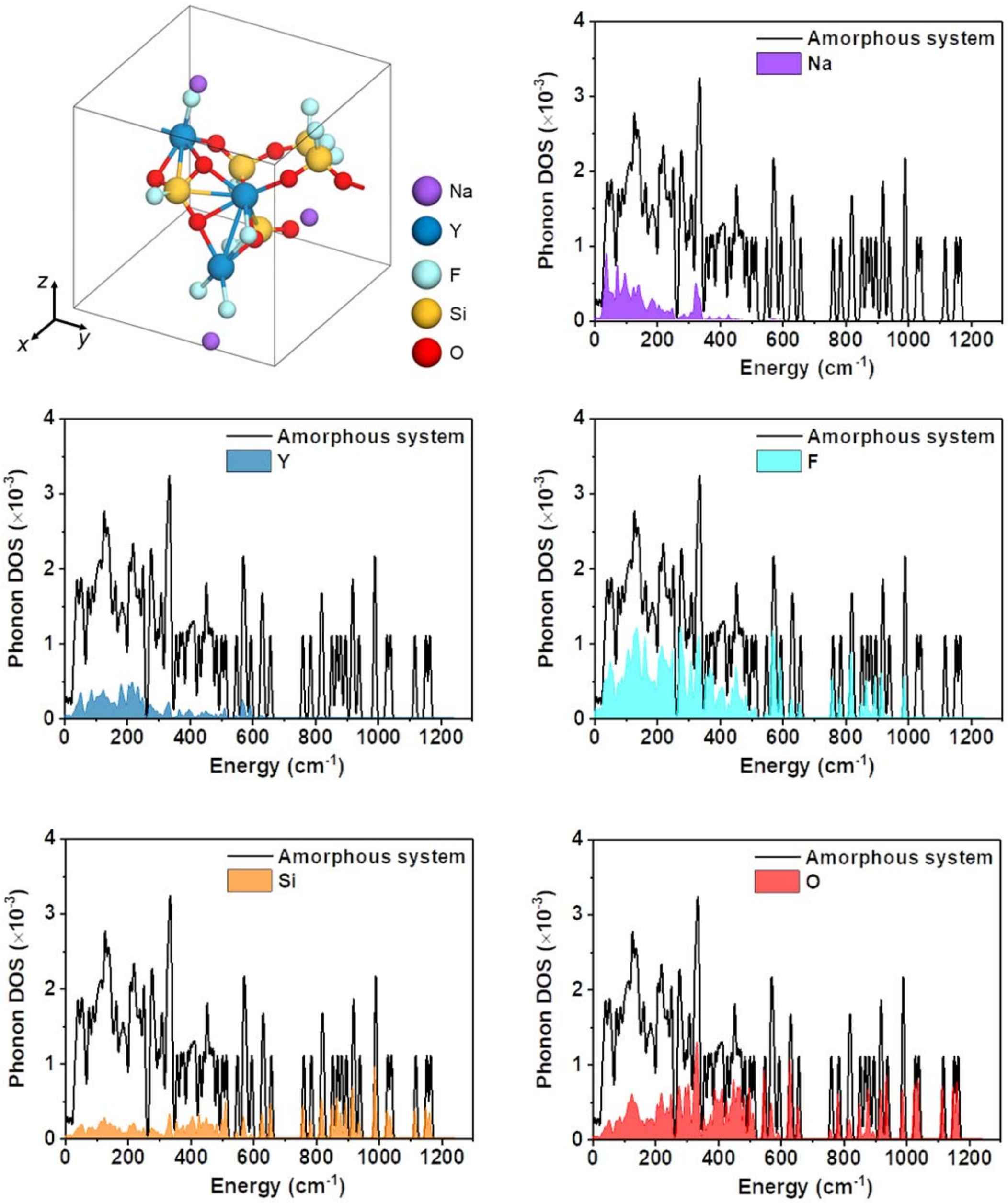

Supplementary Figure 19. Phonon DOS of the amorphous system. Total phonon DOS of the amorphous system and partial phonon DOSs of each element (*i.e.*, Na, Y, F, Si and O atoms) in the amorphous system. Note that the model of the amorphous system is model 2 of Supplementary Figure 11.

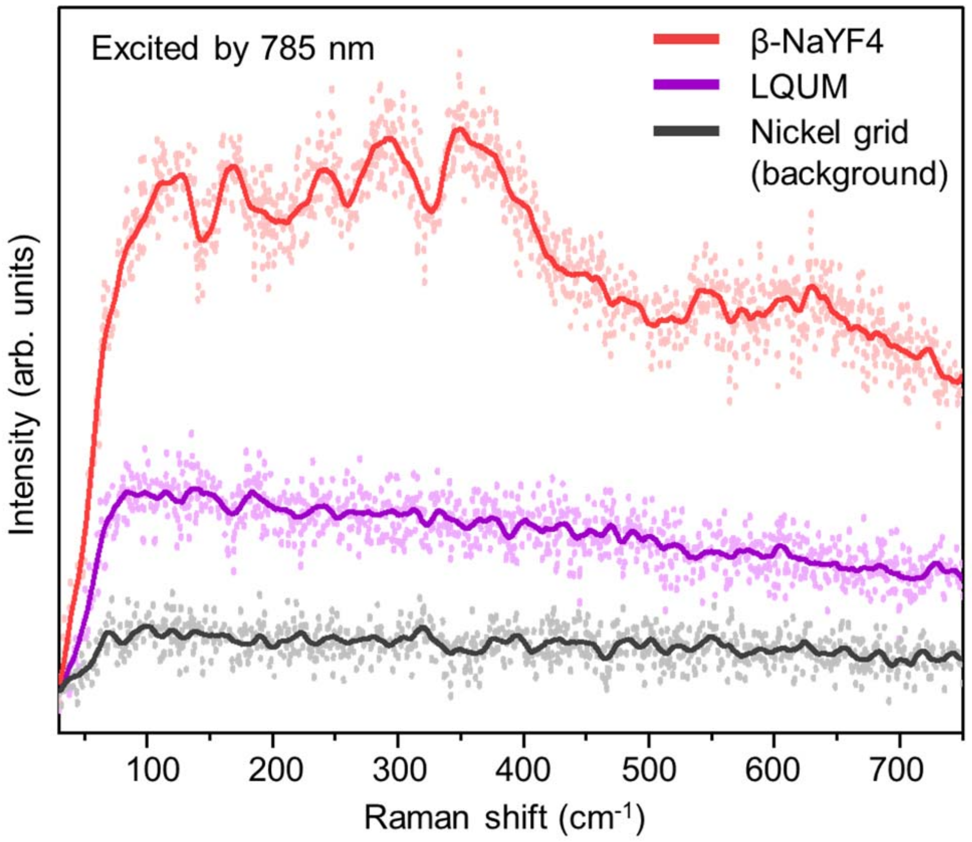

Supplementary Figure 20. Raman spectrum of β-$NaYF_4$, LQUM, and nickel TEM grid (background). To avoid unnecessary excitation of excited states of Er^{3+}, we employed 785 nm laser for Raman spectroscopy (532 nm excites $^4S_{3/2}$, and 633 nm excites $^4F_{9/2}$). For a fair comparison, we investigated each sample on nickel TEM grid and normalize the Raman intensities by sample volume.

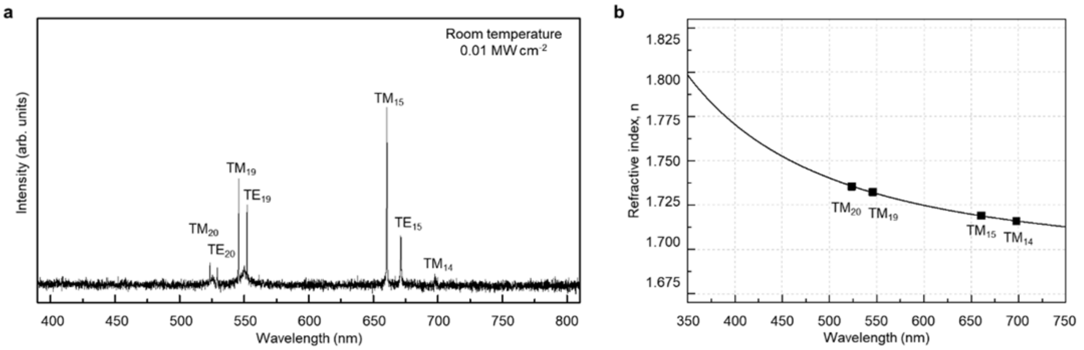

Supplementary Figure 21. Theoretical investigation of laser wavelength. a, The laser emission spectrum of an LQUM under weak excitation (0.01 MW cm^{-2}) at room temperature was used to investigate the coldest pumping conditions. **b**, Refractive index (n) curves were calculated from the TM mode lasing wavelengths of **a**.

Because a small sample volume restricts refractometry measurements, we were not able to directly measure the refractive index of the LQUM. Nevertheless, by virtue of its sparse and simple lasing spectrum (only the first-order mode is present), the refractive index was approximated from the laser emission spectrum. We used the laser emission spectrum obtained under the coldest pumping conditions (Supplementary Figure 21a) to provide the precise radius of the LQUM, which was determined by SEM imaging (2.44 μm). Using asymptotic solutions[18], the integer mode numbers of the TM mode laser peaks were determined to be 14~20 by using a constant refractive index value of 1.73 with the least-squares regression method. However, under real conditions, because the refractive index varies with the wavelength (*i.e.,* it is not constant), we recalculated the refractive index at the given wavelengths using the asymptotic solutions with the determined mode numbers (marked in Supplementary Figure 21b). From the recalculated refractive indices, we could fit the refractive index curve (n) using the one-term Sellmeier equation[19] (one of the simplest equations with high accuracy in the visible range for many oxides and silicates);

$$\frac{1}{n^2 - 1} = -\frac{A}{\lambda^2} + B$$

where parameter A gives the dispersion of the material and parameter B predicts the refractive index at infinite wavelength, $\lambda = \infty$. As a result, we could calculate the one-term Sellmeier parameters of the LQUM: A = 109.1×10^{-16} m^2 and B = 0.5366. The dispersion (*i.e.,* parameter A) of the LQUM is unusually high, as high as those of transition metal compounds (>80 $\times10^{-16}$ m^2) and higher than those of conventional upconversion host materials[20]. Such high dispersion supports the high refractive index of the LQUM[21]. Notably, the refractive index of the LQUM (~1.73) being higher than the refractive indices of the initial materials, β-$NaYF_4$ (~1.48)[22] and amorphous silica (~1.45)[23], is advantageous for light confinement in an optical cavity[24], which contributes to the successful miniaturization of the upconversion microlaser.

Supplementary Note 3

Computational details

Investigation of WGM in upconversion microsphere

To investigate the WGM of our upconversion microsphere, we performed finite element method (FEM) based simulation. In this simulation, the two dimensional (2D) axisymmetric and the wave optics module (electromagnetic waves and frequency domain) are used. The 2D axisymmetric method is a quasi-three dimensional simulation with a certain azimuthal mode number. The extremely fine setting of mesh is adopted with electromagnetic waves and frequency domain contributor. The azimuthal mode numbers are set as 20, 19, and 15 for 525 nm, 550 nm, and 665 nm lasing emission wavelengths, respectively. The refractive index of upconversion microsphere is obtained from the Supplementary Figure 21. The ambient medium is air with 5 μm thickness. The perfect matched layer (PML) is adopted with 1 μm thickness. The refractive index of PML is also air condition. The size of upconversion microsphere is 2.44 μm. The detailed model description is in Supplementary Figure 22a. All simulations were performed using COMSOL Multiphysics software (ver. 5.4).

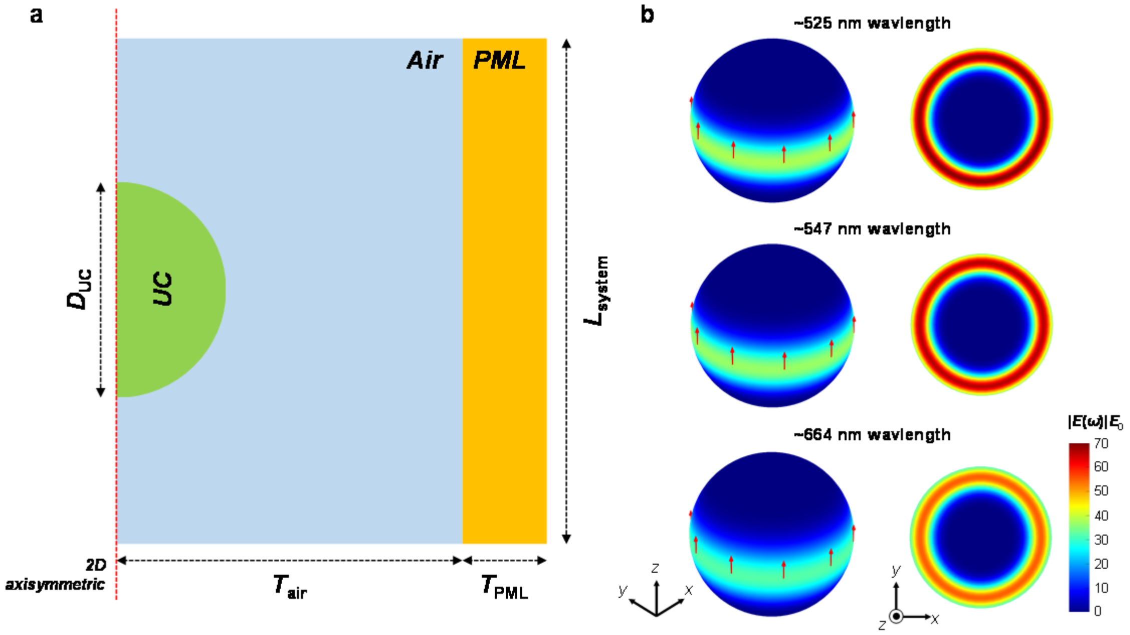

Supplementary Figure 22. WGM simulation of upconversion microsphere. a. Model system for WGM simulation of upconversion microsphere. 2D axisymmetric simulation is performed. Diameter of upconverion microsphere (D_{UC}), thickness of air region (T_{air}), thickness of perfect matched layer (T_{PML}), and length of system (L_{system}) are 2.44, 5, 1, and 10 µm, respectively. **b.** Electric field distributions of upconversion microsphere. Two types of distribution are shown; 3D and 2D cross section distributions. Note that red colored arrows indicate the directions of magnetic field, which describe the TM modes.

To investigate the WGM of our upconversion microsphere, we performed the FEM based simulation (Supplementary Note 3 and Supplementary Figure 22a). In Supplementary Figure 22b, we found that the WGMs of our upconversion microsphere with TM modes (*i.e.*, TM_{20}, TM_{19}, and TM_{15}) were clearly shown around the intense lasing emission wavelengths (*i.e.*, 525 nm, 550 nm, and 665 nm). Accordingly, due to the matching with the WGMs and upconverted emission wavelengths, our upconversion microlaser can be well activated.

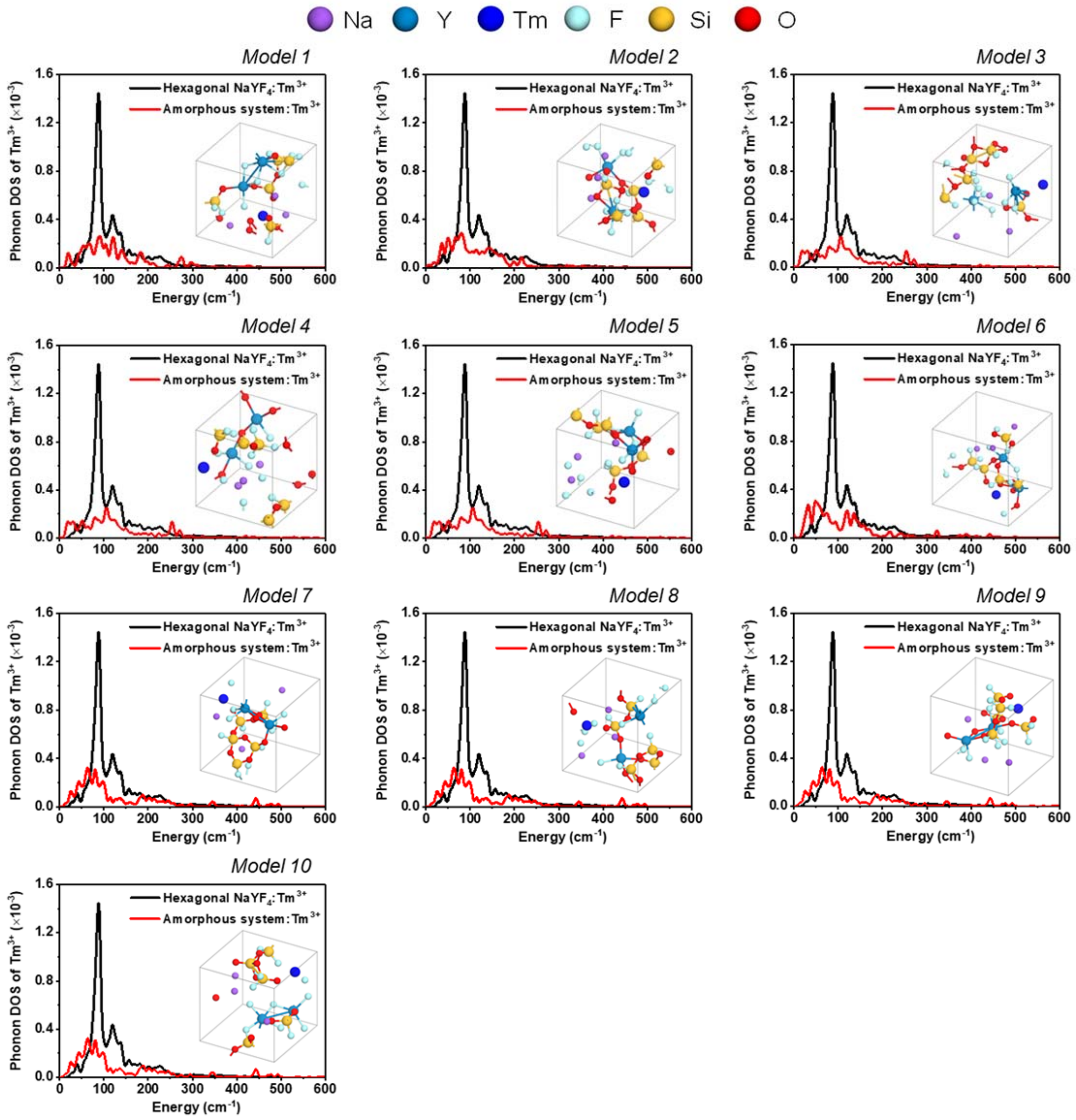

Supplementary Figure 23. Phonon DOS of Tm^{3+} in Tm-doped hexagonal crystal $NaYF_4$ and Tm-doped amorphous systems. Note that the models of Tm-doped amorphous systems and Tm-doped hexagonal crystal $NaYF_4$ are shown in Supplementary Figure 14 and Supplementary Figure 15d, respectively.

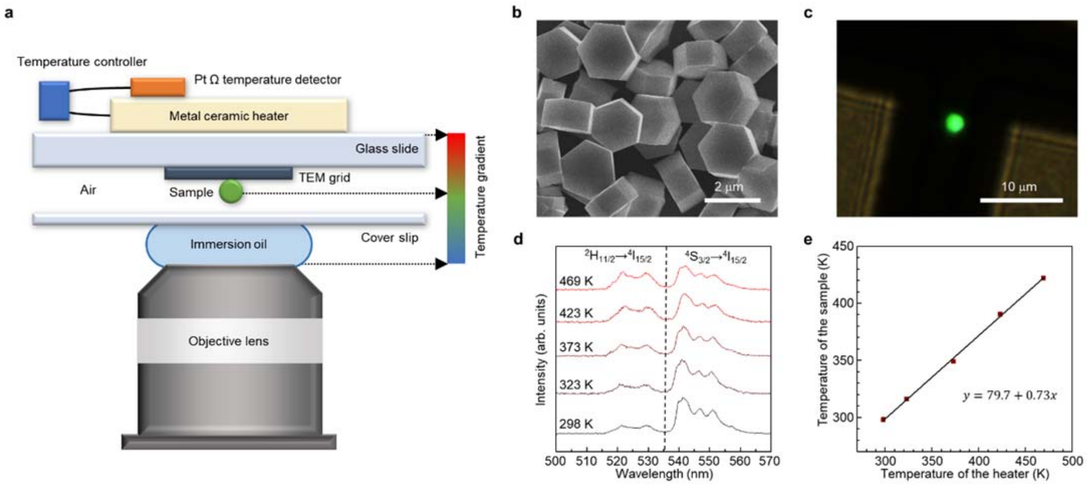

Supplementary Figure 24. Microscopic heating of a sample. a. Schematic of the home-built heating system. **b-c**. SEM (**b**) and color (**c**) images of upconversion microparticles used for temperature calibration. **d**. Emission spectrum of the upconversion microparticles as a function of the metal ceramic heater temperature. **e**. Calibrated temperature obtained using the intensity ratio $^2H_{11/2}/^4S_{3/2}$ ($\rightarrow ^4I_{15/2}$) of the upconversion microparticles.

The home-built heating system for the microscopic sample was operated by a temperature controller (Thorlabs, TC200) via feedback process using a metal ceramic heater (Thorlabs, HT19R) and a temperature detector (Thorlabs, TH100PT) (Supplementary Figure 24a). To adjust the inevitable temperature gradient between the heater and samples, we conducted temperature calibration via an upconversion temperature sensor[25] using β-$NaYF_4$:Yb^{3+}, Er^{3+} (20%, 2%) microparticles. The β-$NaYF_4$:Yb^{3+}, Er^{3+} microparticles were synthesized by a hydrothermal method as previously reported[26]. As-synthesized upconversion microparticles (Supplementary Figure 24b) were deposited on a TEM grid and excited by a CW 980-nm pump laser (Supplementary Figure 24c). Supplementary Figure 20d shows the upconversion spectrum of β-$NaYF_4$:Yb^{3+}, Er^{3+} (20%, 2%) microparticles at various heater temperatures. Given that the intensity ratio of $^2H_{11/2}\rightarrow ^4I_{15/2}$ to $^4S_{3/2}\rightarrow ^4I_{15/2}$ (R_{HS}) is dominated by a Boltzmann thermal distribution[27], the temperature of the sample in the microscopic heating system can be estimated by using the equation $R_{HS} = \alpha exp(-\Delta E/k_bT)$, where ΔE is the

energy separation between the $^2H_{11/2}$ and $^4S_{3/2}$ states (~800 cm^{-1}), k_b is Boltzmann's constant and T is the absolute temperature. The surrounding temperature of the microscopic sample was calibrated as shown in Supplementary Figure 24e using the constant α, which is determined by a reference value at room temperature.

Supplementary References

1 Moon, B. S., Kim, H. E. & Kim, D. H. Ultrafast Single-Band Upconversion Luminescence in a Liquid-Quenched Amorphous Matrix. *Adv. Mater.* **30**, e1800008 (2018).

2 Humar, M. & Yun, S. H. Intracellular microlasers. *Nat. Photonics* **9**, 572-576 (2015).

3 Kippenberg, T. J. A. Nonlinear optics in ultra-high Q whispering-gallery optical microcavities, Doctoral dissertation, California Institute of Technology (2004).

4 Gu, F. *et al.* Single whispering-gallery mode lasing in polymer bottle microresonators via spatial pump engineering. *Light Sci. Appl.* **6**, e17061 (2017).

5 Ankudinov, A. V. *et al.* Investigation of the light field of a semiconductor diode laser. *Opt. Express* **22**, 26438-26448 (2014).

6 Sun, H. in Laser Diode Beam Basics, Manipulations and Characterizations Springer Briefs in Physics, Chapter 2, 21-37 (2012).

7 Balac, S. & Féron, P. Whispering gallery modes volume computation in optical micro-spheres, Doctoral dissertation, FOTON, UMR CNRS 6082, (2014)

8 Rappé, A. K., Casewit, C. J., Colwell, K., Goddard III, W. A. & Skiff, W. M. UFF, a full periodic table force field for molecular mechanics and molecular dynamics simulations. *J. Am. Chem.* **114**, 10024-10035 (1992).

9 Nosé, S. A molecular dynamics method for simulations in the canonical ensemble. *Mol. Phys.* **52**, 255-268 (1984).

10 Materials Studio 2019, BIOVIA Inc, San Diego, CA, 2019.

11 Clark, S. J. *et al.* First principles methods using CASTEP. *Z. Kristallogr. Cryst. Mater.* **220**, 567-570 (2005).

12 Perdew, J. P., Burke, K. & Ernzerhof, M. Generalized gradient approximation made

simple. *Phys. Rev. Lett.* **77**, 3865 (1996).

13 Kleinman, L. & Bylander, D. M. Efficacious Form for Model Pseudopotentials. *Phys. Rev. Lett.* **48**, 1425-1428 (1982).

14 Monkhorst, H. J. & Pack, J. D. Special points for Brillouin-zone integrations. *Phys. Rev. B* **13**, 5188-5192.

15 Andersen, H. C. Molecular dynamics simulations at constant pressure and/or temperature. *J. Chem. Phys.* **72**, 2384-2393 (1980).

16 Montanari, B. & Harrison, N. Lattice dynamics of TiO2 rutile: influence of gradient corrections in density functional calculations. *Chem. Phys. Lett.* **364**, 528-534 (2002).

17 Pfrommer, B. G., Côté, M., Louie, S. G. & Cohen, M. L. Relaxation of crystals with the quasi-Newton method. *J. Comput. Phys.* **131**, 233-240 (1997).

18 Tang, S. K. Y., Derda, R., Quan, Q., Lončar, M. & Whitesides, G. M. Continuously tunable microdroplet-laser in a microfluidic channel. *Opt. Express* **19**, 2204-2215 (2011).

19 Medenbach, O. & Shannon, R. Refractive indices and optical dispersion of 103 synthetic and mineral oxides and silicates measured by a small-prism technique. *JOSA B* **14**, 3299-3318 (1997).

20 D. Shannon, R., Shannon, R. C., Medenbach, O. & Fischer, R. X. Refractive index and dispersion of fluorides and oxides. *J. Phys. Chem. Ref. Data* **31**, 931-970 (2002).

21 Simmrock, H.-U., Mathy, A., Dominguez, L., Meyer, W. H. & Wegner, G. Polymers with a High Refractive Index and Low Optical Dispersion. *Angew. Chem.* **101**, 1148-1149 (1989).

22 Sokolov, V. I. *et al.* Determination of the refractive index of β-$NaYF_4/Yb^{3+}/Er^{3+}/Tm^{3+}$ nanocrystals using spectroscopic refractometry. *Opt. Spectrosc.* **118**, 609-613 (2015).

23 Velikov, K. P. & van Blaaderen, A. Synthesis and Characterization of Monodisperse Core−Shell Colloidal Spheres of Zinc Sulfide and Silica. *Langmuir* **17**, 4779-4786 (2001).

24 He, L., Özdemir, Ş. K. & Yang, L. Whispering gallery microcavity lasers. *Laser Photonics Rev.* **7**, 60-82 (2013).

25 Berthou, H. & Jörgensen, C. K. Optical-fiber temperature sensor based on upconversion-excited fluorescence. *Opt. Lett.* **15**, 1100-1102 (1990).

26 Yao, W. *et al.* Large-scale synthesis and screen printing of upconversion hexagonal-phase $NaYF_4$:Yb^{3+},Tm^{3+}/Er^{3+}/Eu^{3+} plates for security applications. *J. Mater. Chem. C* **4**, 6327-6335 (2016).

27 Yu, W., Xu, W., Song, H. & Zhang, S. Temperature-dependent upconversion luminescence and dynamics of $NaYF_4$:Yb^{3+}/Er^{3+} nanocrystals: influence of particle size and crystalline phase. *Dalton Trans.* **43**, 6139-6147 (2014).
